# Stone removal by percutaneous papillary balloon dilatation for cystic duct and bile duct stones after cholecystectomy and distal gastrectomy with Roux-en-Y gastrojejunostomy

**DOI:** 10.1016/j.radcr.2022.09.078

**Published:** 2022-10-28

**Authors:** Fumio Chikamori, Koji Ueta, Kazuhisa Onishi, Mitsuteru Yoshida, Nobuyuki Tanida, Hiromichi Yamai, Hisashi Matsuoka, Norihiro Hokimoto, Jun Iwabu, Ryo Yamada, Kai Mizobuchi, Shigeto Shimizu, Niranjan Sharma

**Affiliations:** a Department of Surgery, Japanese Red Cross Kochi Hospital, 1-4-63-11 Hadaminamimachi, Kochi, 780-8562, Japan; b Adv Train Gastroint & Organ Transp Surgery, 12 Scotland St, Dunedin, 9016, New Zealand

**Keywords:** Percutaneous papillary balloon dilatation, Percutaneous stone removal, Cystic duct stone, Roux-en-Y gastrojejunostomy, Cobra-shaped sheath, PPBD, percutaneous papillary balloon dilatation, PTBD, percutaneous transhepatic biliary drainage, MRI, magnetic resonance imaging, MRCP, magnetic resonance cholangiopancreatography, EHL, electrohydraulic lithotripsy

## Abstract

A 71-year-old woman was referred to our department for abdominal pain. She was diagnosed with acute obstructive cholangitis due to cystic duct and bile duct stones after cholecystectomy and Roux-en-Y gastrojejunostomy. Two years ago, the patient underwent endoscopic and laparoscopic treatment for cystic duct and bile duct stones, however, the stones remained. This time, she was treated with stone removal using percutaneous papillary balloon dilatation (PPBD). Large stones in the common hepatic and bile ducts were crushed by electrohydraulic lithotripsy and then pushed out into the duodenum through the dilated papilla of Vater using a balloon catheter covered with the sheath and cholangioscopy. Stone in the cystic duct was pulled to the common bile duct and pushed to the duodenum. Stone removal using PPBD is an excellent alternative for patients with cystic duct and bile duct stones unable to be treated with endoscopic or laparoscopic stone removal.

## Introduction

Endoscopic treatment is now widely used as the first choice for bile duct stones [1], [2], [3], [4]. With advances in endoscopic technology, endoscopists are actively challenging bile duct stone removal in patients who have undergone Roux-en-Y reconstruction or Billroth's operation II [5,6]. However, in clinical practice, there are some cases with bile duct stones that are still difficult to treat [7]. There is no established next-line treatment after endoscopic removal of bile duct stones fails. Here, we report a case of the cystic duct and bile duct stones after cholecystectomy and Roux-en-Y gastrojejunostomy that was treated with less invasive stone removal by percutaneous papillary balloon dilatation (PPBD) [8,9] using a cobra-shaped sheath and cholangioscopy.

## Case report

A 71-year-old woman was referred to our department with abdominal pain and jaundice. She had surgery for early gastric cancer and cholecystolithiasis at the age of 67. At that time, she underwent cholecystectomy, distal gastrectomy, and Roux-en-Y gastrojejunostomy.

At the age of 69, she suffered from acute obstructive cholangitis due to cystic duct and bile duct stones. Peroral endoscopic stone removal was attempted but failed. So, she underwent laparoscopic choledochotomy and T-tube bile drainage. One month after surgery, the papilla of Vater was dilated to 11 mm in diameter by endoscopic papillary balloon dilatation using the rendezvous technique. Two months after the removal of the T-tube, residual stones were found. Peroral endoscopic stone removal was retried but failed again. After that, she was followed up at our hospital.

On admission, her temperature was 38.2°C and her blood pressure was 141/85 mmHg. Her height was 160 cm, body weight was 41.3kg, and body mass index was 16.1 kg/m^2^. She had tenderness in her right hypochondrium. Laboratory results showed hemoglobin 12.6 g/dL (normal range, 11.0-14.6), white blood cell count 3900 /µL (3500-8000), platelet count 13.2 × 10^4^ /µL (12.3-33.1), total bilirubin 5.8 mg/dL (0.3-1.3), alanine aminotransaminase 239 IU/L (10-32), aspartate aminotransferase 560 IU/L (5-27), alkaline phosphatase 242 U/L (38-113), γ-glutamyl transpeptidase 166 U/L (11-64), serum amylase 738 IU/L(28-99), blood urea nitrogen 12.2 mg/dL (8-20), creatinine 0.55 mg/dL (0.36-1.06), prothrombin time (PT) 12.6 sec (9-12), PT% 84.1% (70-130), international normalized ratio 1.1, activated partial thromboplastin time 23.7 sec (20-35), procalcitonin 5.56 ng/mL (0-0.49), C-reactive protein 11.97 mg/dL (<0.16), and brain natriuretic peptide 23.4 ng/mL (0-18.4). Based on the clinical findings and laboratory workup, acute obstructive cholangitis with sepsis was suspected and was treated with a systemic antibiotic.

**Fig. 1:**
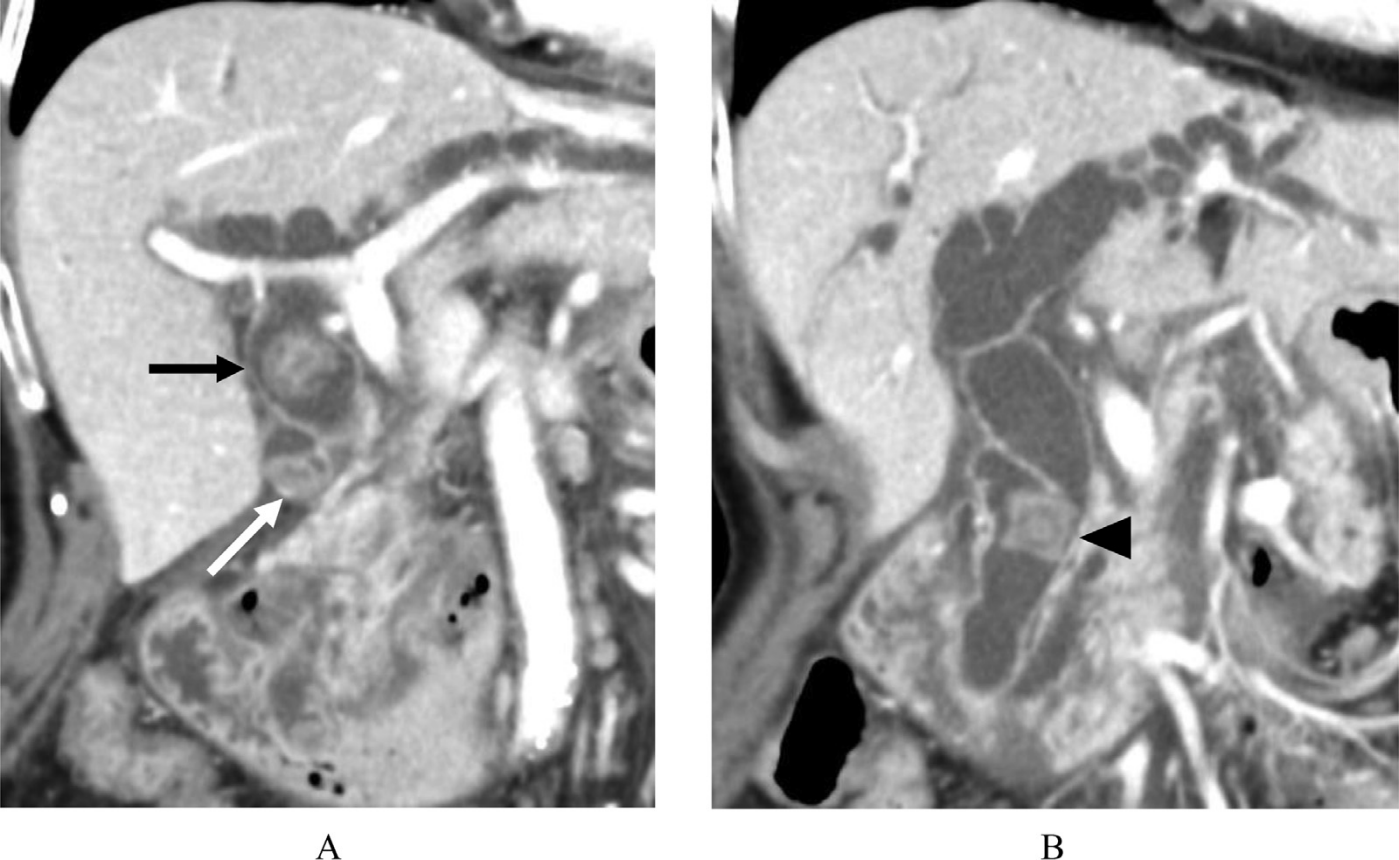
(A) Coronal image of abdominal contrast-enhanced CT shows common hepatic duct stone (black arrow) and cystic duct stone (white arrow). (B) Another coronal image of abdominal contrast-enhanced CT shows common bile duct stone (arrowhead).

**Fig. 2:**
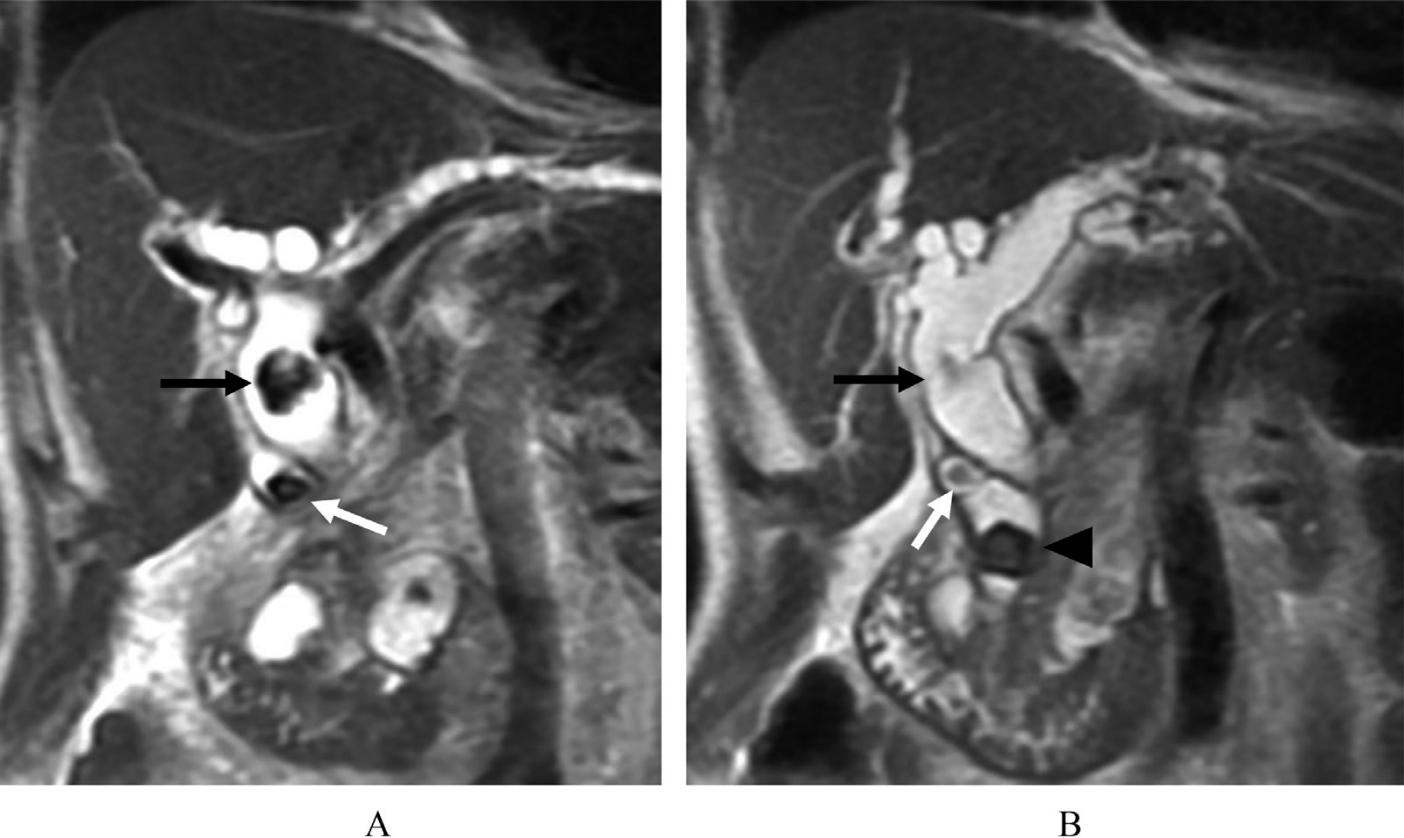
(A) Coronal image of T2-weighted MRI shows common hepatic duct stone (black arrow) and cystic duct stone (white arrow). (B) Another coronal image of T2-weighted MRI shows cystic duct stone (white arrow) and common bile duct stone (arrowhead).

Abdominal ultrasonography (US) showed high-echoic stones in the dilated bile duct. Contrast-enhanced abdominal computed tomography (CT) showed multiple stones in the cystic duct, common hepatic duct, and common bile duct with biliary dilatation and duodenal parapapillary diverticulums (Figs. 1A and B). The abdominal magnetic resonance imaging (MRI) and magnetic resonance cholangiopancreatography (MRCP) also showed multiple stones of varying sizes (8, 17, and 14mm, respectively) in the cystic duct, common hepatic duct, and common bile duct (Figs. 2A and B).

**Fig. 3:**
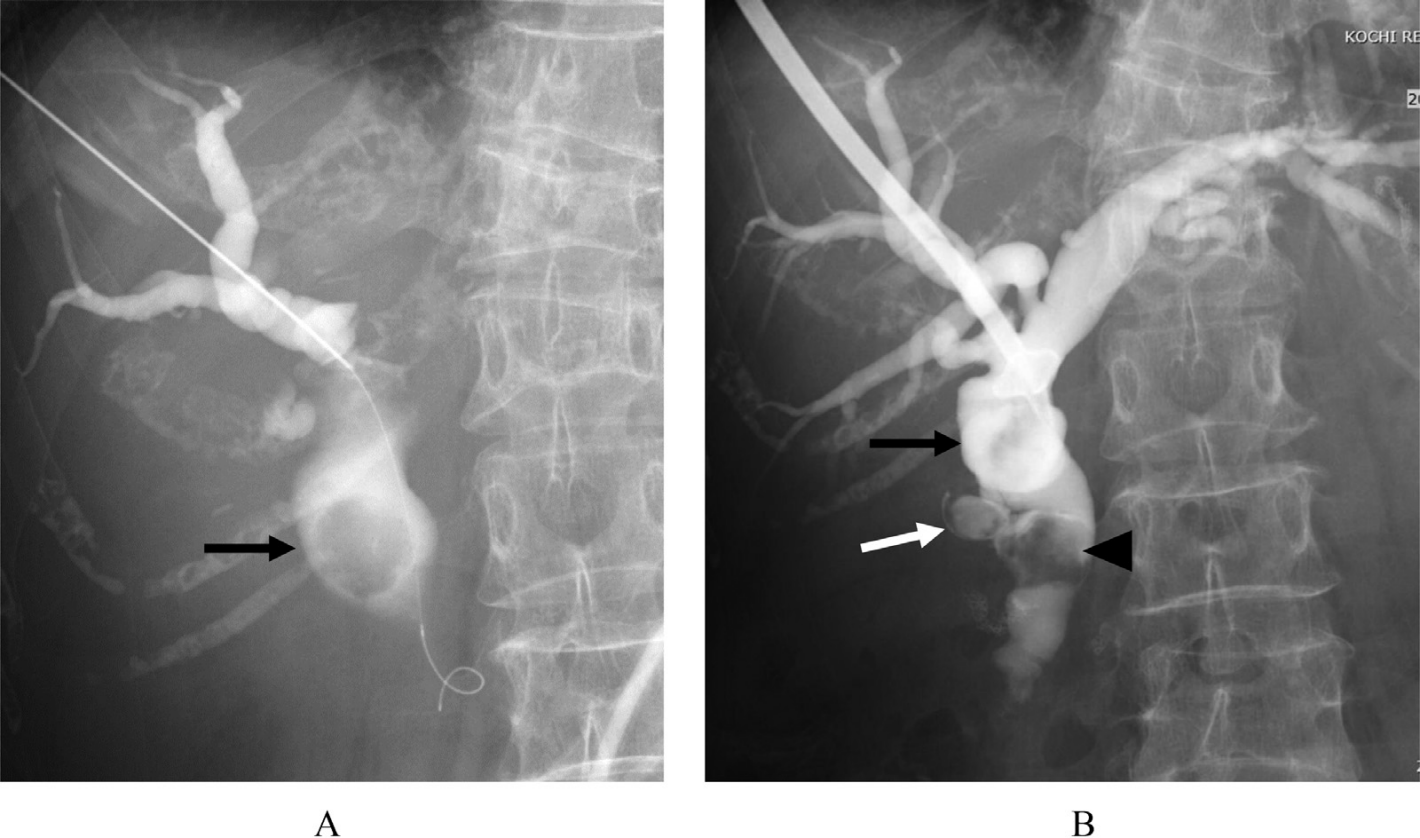
(A) Initial percutaneous transhepatic cholangiogram shows a common hepatic large stone (black arrow). (B) The second cholangiogram via PTBD tube shows multiple stones in the cystic duct (white arrow), common hepatic duct (black arrow), and common bile duct (arrowhead).

Percutaneous transhepatic biliary drainage (PTBD) was planned on the 2nd day of hospitalization. Before PTBD, color Doppler US was performed to avoid vessel puncture. Initial percutaneous transhepatic cholangiography through the right anterior hepatic duct revealed a large stone in the common hepatic duct (Fig. 3A). After introducing a guidewire into the bile duct through the puncture needle, a 10 Fr. PTBD tube was inserted into the common hepatic duct using a 2-step method. A second cholangiogram through the PTBD tube revealed multiple stones in the cystic duct, common hepatic duct, and common bile duct (Fig. 3B). The culture of bile showed the presence of *Escherichia coli, Klebsiella oxytoca*, and *Enterococcus raffinosus*. The culture of blood showed the presence of *E coli*, and *Klebsiella pneumonia* sensitive to a given antibiotic.

**Fig. 4:**
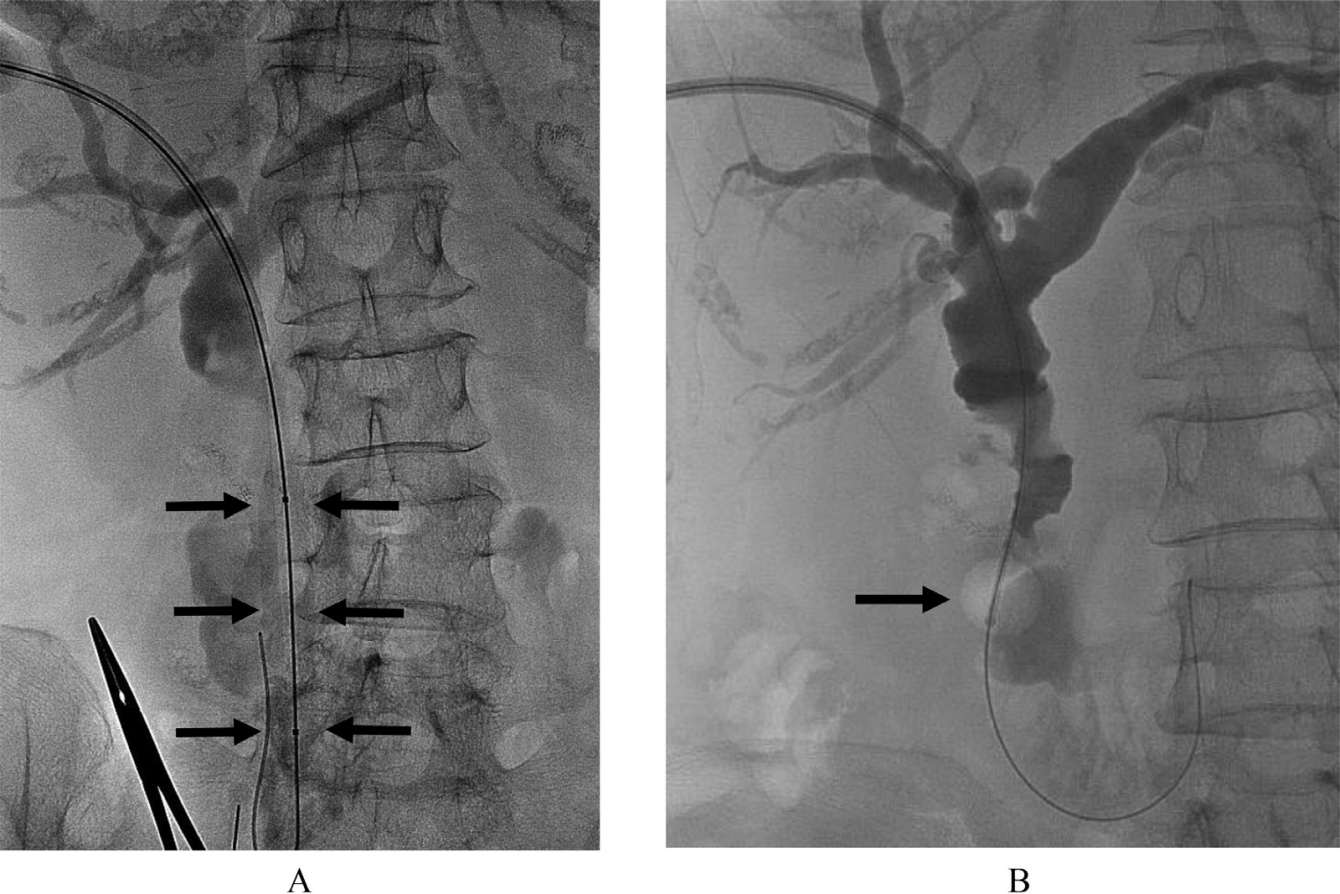
(A) Balloon dilation of the papilla of Vater (black arrow). (B) Cholangiogram shows a removal balloon catheter (black arrow) covered with the sheath that pushes stones into the duodenum.

**Fig. 5:**
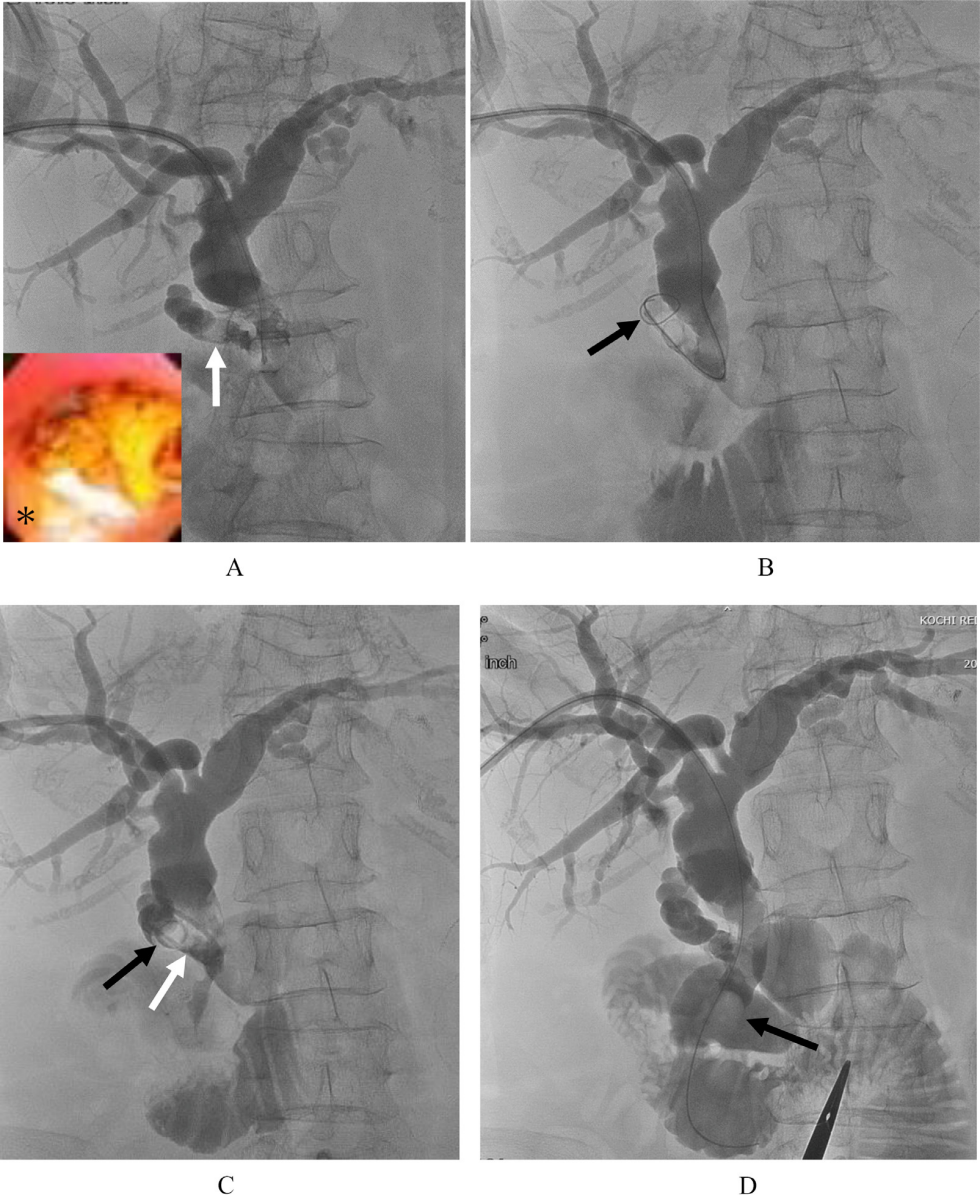
(A) Cholangiogram shows cystic duct stone (white arrow). (A*) Cholangioscopic picture shows cystic duct stone. (B) Cholangiogram shows a 5 Fr. balloon catheter and guidewire in the cystic duct (black arrow). (C) Cholangiogram shows that the balloon (black arrow) is inflated and the stone (white arrow) is pulled into the common bile duct. (D) Balloon-occluded cholangiogram using cobra-shaped sheath and 6Fr. balloon catheter shows clearance of cystic duct, common hepatic, and common bile duct stones. The contrast medium is injected through the sheath. An inflated balloon to occlude the dilated papilla of Vater is indicated by an arrow.

**Fig. 6:**
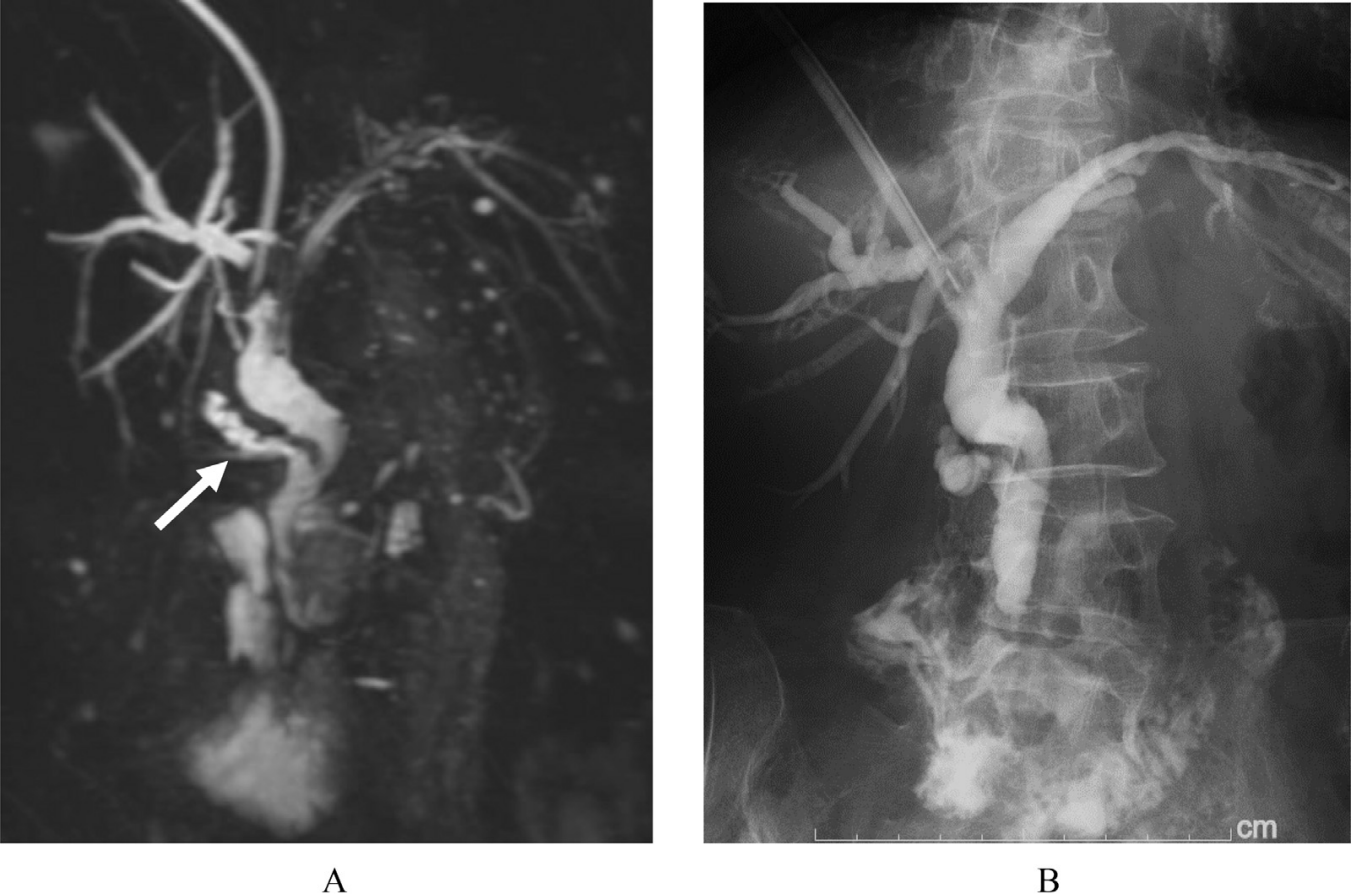
(A) MRCP 10 days after percutaneous stone removal shows no residual stone in the cystic duct (white arrow). (B) Repeated and final cholangiogram 10 days after percutaneous stone removal shows that the cystic duct, common hepatic, and common bile ducts are free of stones.

Two days after PTBD, alkaline phosphatase, an indicator of cholestasis, normalized to 103 U/L. Ten days after PTBD, the cholangitis was relieved, and the patient was temporarily discharged with the PTBD tube left in situ. She was readmitted 5 weeks after PTBD, and the PTBD route was dilated to 18 Fr. Six weeks after PTBD, she was treated by stone removal with PPBD using an 8 Fr. cobra-shaped sheath and cholangioscopy. The papilla of Vater was dilated with a balloon 12 mm in diameter for 10 minutes at 2 atm (Fig. 4A). Common hepatic and bile ducts stones were crushed by electrohydraulic lithotripsy (EHL) and then pushed out into the duodenum through the dilated papilla of Vater using a 6 Fr. balloon catheter covered with the sheath and cholangioscopy (Fig. 4B). The cystic duct stone was observable with a cholangioscope (Figs. 5A and A*), but the bending of the cholangioscope made it difficult to insert the EHL probe into the optimal position. For stone located in the cystic duct, a 5 Fr. balloon catheter was inserted into the cystic duct following a guidewire by using the cobra-shaped sheath (Fig. 5B). The balloon was inflated and the stone was pulled into the common bile duct (Fig. 5C). Then the stone was pushed out into the duodenum. Clearance of cystic duct and bile duct stones was confirmed by balloon-occluded cholangiography using a cobra-shaped sheath and a 6 Fr. balloon catheter (Fig. 5D). After that, cholangiomanometry was performed. The resistance and residual biliary pressure values at zero flow rate were 5 units (normal range, 1-7) and 11.5 cmH20 (normal range, 5-15), respectively. These values were not considered abnormal by the Nagakawa criteria [10]. Infrared spectroscopy revealed that more than 95% of the bile duct stone component was bilirubin calcium. She was discharged 3 days after percutaneous stone removal. Ten days after percutaneous stone removal, the PTBD tube was removed after confirming that the cystic duct, common hepatic, and common bile ducts were free of stones on MRCP and final cholangiography (Figs. 6A and B).

## Discussion

We reported a case of multiple stones in the cystic duct, common hepatic, and common bile ducts after cholecystectomy and Roux-en-Y gastrojejunostomy, that was successfully treated by stone removal using PPBD and EHL. Two years ago, the patient was treated with endoscopic and laparoscopic removal; however, stones remained and worsened. In this case, incomplete stone clearance [11,12] after endoscopic and laparoscopic removal is considered to be the main cause of stone reformation and cholangitis. There were 3 important issues in this case: (1) Which treatment should be selected next when endoscopic stone removal is difficult for bile duct stones with Roux-en-Y gastrojejunostomy? (2) What should be the treatment choice for remnant cystic duct stone after cholecystectomy? (3) How to confirm a stone-free state after bile duct stone treatment?

Endoscopic stone removal is an effective and less invasive technique for common bile duct stones. Bile duct stones in Roux-en-Y reconstruction cases have become treatable with the application of balloon endoscopy [5,6], but there are still cases where stone removal is difficult [7]. Even in the usual cases of bile duct stones without Roux-en-Y reconstruction, the recurrence rate of bile duct stones after endoscopic stone removal has been reported 4% - 24%. The incidence of residual stones after mechanical lithotripsy for intractable bile duct stones is 24%-40% [1]. In clinical practice, recurrent cholangitis due to residual bile duct stones occurs frequently even after endoscopic stone removal [2].

There are some next treatment options when endoscopic stone removal is difficult for bile duct stones in post-Rou-en-Y gastrojejunostomy cases: laparoscopic choledochotomy [13,14], open choledocotomy, endoscopic ultrasonography-guided procedure [15,16], rendezvous technique [17,18], PPBD [8,9], and percutaneous transhepatic choledochoscopic lithotomy [19], etc. Each method has advantages and disadvantages, and each facility tends to select the method that they are familiar with. If we pursue a minimally invasive concept, we consider that percutaneous stone removal using PPBD should be the next step when endoscopic removal fails for bile duct stones after Roux-en Y reconstruction with cholecystectomy as in this case.

Some institutions choose laparoscopic choledochotomy when endoscopic treatment for bile duct stones after cholecystectomy fails. However, laparoscopic stone removal takes time due to adhesion in postoperative cases. There is also the problem of contamination of the abdominal cavity with bile. With laparoscopic surgery, it is uncertain if multiple stones can be completely removed. The postoperative rendezvous technique using T-tube or C-tube route is one of the means to solve this problem. However, in fact, in this case, this procedure was attempted 2 years ago, but the stones remained. The presence of these residual stones caused stone growth over 2 years. For these reasons, we chose percutaneous stone removal this time.

As long as the PTBD route is secured, PPBD is technically less difficult than endoscopic removal, especially in Rox-en-Y cases. Intrahepatic vascular injury due to percutaneous puncture in PTBD can be avoided by color Doppler-guided procedure and the 2-step method. This time, the PTBD route was dilated and cholangioscopy and EHL were used because the common hepatic duct stone was as large as 17 mm in diameter. If the stone size is less than 12mm, it can be treated with the usual PPBD through an 8 Fr. cobra-shaped sheath without dilatation of the PTBD route. The sheath was developed for the treatment of gastric varices [20]. Its use in PPBD for cholecytocholedocholithiasis has been reported [8,9]. The sheath contributes to ensuring the operability and strength of the balloon catheter in stone removal. Recently, we could successfully treat a case of multiple hepatolithiasis with choledochoduodenal anastomotic stenosis by using this sheath [7]. The sheath was also useful for guiding the stone removal catheter to the periphery of the bile duct.

In the percutaneous approach, the stone removal procedure is performed close to the hand, so the force is easily transmitted. The procedure can be easily repeated. Therefore, percutaneous intervention can be of sufficient value between endoscopic and open surgery in cases of multiple bile duct stones or postoperative cases. Although the PTBD tube period will be longer, as in this case, outpatient follow-up can be performed after cholangitis has subsided. PPBD itself has a short hospital stay. In this case, the patient was discharged 3 days after PPBD.

The most important concern was whether the remnant cystic duct stone [21] is successfully removed. In the era of laparoscopic cholecystectomy, there is a tendency to leave a long cystic duct stump without intraoperative cholangiography. Palanivelu et al. [22] reported an incidence of remnant cystic duct stones was 4.19% in patients undergoing laparoscopic cholecystectomy as compared to an incidence of 0.02% in patients undergoing conventional open cholecystectomy. The feasibility of cystic duct stone removal by percutaneous approach seems to depend on the confluence angle and the length of the cystic duct. The percutaneous approach may be inferior to endoscopic approaches at this point. However, in this case, the guidewire was deeply inserted into the remnant cystic duct with a U-turn. Then, through the sheath, a 5 Fr. balloon catheter was inserted along the guidewire and the stone could be removed by traction

Preservation of the function of the papilla of Vater is considered useful for suppressing the recurrence of bile duct stones [10]. In this case, the papilla of Vater was dilated to 12 mm, but the cholangiomanometry was confirmed to be normal. In addition, we were able to achieve a stone-free state with percutaneous treatment, which was not possible with endoscopic and laparoscopic treatment in the past.

In order to verify whether or not the patient was stone-free after the treatment, we used cholangioscopic observation of the intra and extrahepatic bile ducts, cholaniomanometry, repeated cholangiography including balloon occluded cholangiography, and MRCP. In endoscopic or laparoscopic removal, post-treatment cholangiography sometimes poses a problem in distinguishing between air and stones. During stone removal, air enters the bile duct, reducing the reliability of cholangiography. Therefore, it is preferable to perform cholangiography at a later date to confirm the presence or absence of residual stones. The percutaneous approach has a PTBD route, so thorough and repeated cholangiography is possible and easy. If residual stones are suspected, repeated PPBD can easily be added.

It is important to make a stone-free state for intractable bile duct stones to suppress recurrence. Therefore, even in the era of endoscopic and laparoscopic surgery, percutaneous removal of bile duct stones still has great significance. Monitoring liver function is also important during the long-term follow-up after stone removal. We conclude that stone removal using PPBD is an excellent alternative for patients with cystic duct and bile duct stones unable to be treated with endoscopic or laparoscopic stone removal.

## Patient consent

Written informed consent was obtained from the patient for publication of this case report and accompanying images.

## References

[1] Lin YY, Wang YD, Yue P, Zhang XZ, Leung JW, Jiao PP (2021). Could saline irrigation clear all residual common bile duct stones after lithotripsy? A self-controlled prospective cohort study. World J Gastroenterol.

[2] Ahn DW, Lee SH, Paik WH, Song BJ, Park JM, Kim J (2018). Effects of saline irrigation of the bile duct to reduce the rate of residual common bile duct stones: a multicenter, prospective, randomized study. Am J Gastroenterol.

[3] Endo R, Satoh A, Tanaka Y, Shimoda F, Suzuki K, Takahashi K (2020). Saline solution irrigation of the bile duct after stone removal reduces the recurrence of common bile duct stones. Tohoku J Exp Med.

[4] Kim JH, Yang MJ, Hwang JC, Yoo BM. (2013). Endoscopic papillary large balloon dilation for the removal of bile duct stones. World J Gastroenterol.

[5] Itoi T, Ishii K, Sofuni A, Itokawa F, Kurihara T, Tsuchiya T (2011). Large balloon dilatation following endoscopic sphincterotomy using a balloon enteroscope for the bile duct stone extractions in patients with Roux-en-Y anastomosis. Dig Liver Dis.

[6] Obata T, Tsutsumi K, Kato H, Ueki T, Miyamoto K, Yamazaki T (2021). Balloon enteroscopy-assisted endoscopic retrograde cholangiopancreatography for the treatment of common bile duct stones in patients with Roux-en-Y gastrectomy: outcomes and factors affecting complete stone extraction. J Clin Med.

[7] Chikamori F, Shimizu S, Ito S, Okazaki M, Tanida N, Sharma N. (2022). Percutaneous stone removal using cobra-shaped sheath and cholangioscopy for multiple hepatolithiasis with choledochoduodenal anastomotic stenosis. Radiol Case Rep.

[8] Chikamori F, Nishio S, LeMaster JC. (1999). Percutaneous papillary balloon dilatation as a therapeutic option for cholecystocholedocholithiasis in the era of laparoscopic cholecystectomy. Surg Today.

[9] Chikamori F, Kuniyoshi N, Shibuya S, Takase Y. (2003). Simultaneous laparoscopic cholecystectomy and percutaneous papillary balloon dilatation for cholecystocholedocholithiasis. Dig Surg.

[10] Nagakawa T, Minai S, Ueno K, Ohta T, Kayahara M, Akiyama T (1994). Variable loading cholangiomanometry and clinical applications. Hepato-Gastroenterol.

[11] Konstantakis C, Triantos C, Theopistos V, Theocharis G, Maroulis I, Diamantopoulou G (2017). Recurrence of choledocholithiasis following endoscopic bile duct clearance: long term results and factors associated with recurrent bile duct stones. World J Gastrointest Endosc.

[12] Tsuchiya S, Tsuyuguchi T, Sakai Y, Sugiyama H, Miyagawa K, Fukuda Y (2008). Clinical utility of intraductal US to decrease early recurrence rate of common bile duct stones after endoscopic papillotomy. J Gastroenterol Hepatol.

[13] Liu S, Fang C, Tan J, Chen W. (2020). A comparison of the relative safety and efficacy of laparoscopic choledochotomy with primary closure and endoscopic treatment for bile duct stones in patients with cholelithiasis. J Laparoendosc Adv Surg Tech A.

[14] Suwatthanarak T, Akaraviputh T, Phalanusitthepha C, Chinswangwatanakul V, Methasate A, Swangsri J (2021). Outcomes of laparoscopic common bile duct exploration by chopstick technique in choledocholithiasis. JSLS.

[15] James TW, Fan YC, Baron TH. (2018). EUS-guided hepaticoenterostomy as a portal to allow definitive antegrade treatment of benign biliary diseases in patients with surgically altered anatomy. Gastrointest Endosc.

[16] Mukai S, Tsuchiya T, Itoi T. (2019). Interventional endoscopic ultrasonography for benign biliary diseases in patients with surgically altered anatomy. Curr Opin Gastroenterol.

[17] Kimura K, Kudo K, Kurihara T, Yoshiya S, Mano Y, Takeishi K (2019). Rendezvous technique using double balloon endoscope for removal of multiple intrahepatic bile duct stones in hepaticojejunostomy after living donor liver transplant: a case report. Transplant Proc.

[18] Noel R, Arnelo U, Swahn F. (2019). Intraoperative versus postoperative rendezvous endoscopic retrograde cholangiopancreatography to treat common bile duct stones during cholecystectomy. Dig Endosc.

[19] Zhuo H, Chen Z, Lin R, Yang S, Zhuang H, He C (2020). Percutaneous transhepatic choledochoscopic lithotomy (PTCSL) is effective for the treatment of intrahepatic and extrahepatic choledocholithiasis. Surg Laparosc Endosc Percutan Tech.

[20] Chikamori F, Shibuya S, Takase Y, Ozaki A, Fukao K. (1996). Transjugular retrograde obliteration for gastric varices. Abdom Imaging.

[21] Amin A, Zhurov Y, Ibrahim G, Maffei A, Giannone J, Cerabona T (2016). Combined endoscopic and laparoscopic management of postcholecystectomy mirizzi syndrome from a remnant cystic duct stone: case report and review of the literature. Case Rep Surg.

[22] Palanivelu C, Rangarajan M, Jategaonkar PA, Madankumar MV, Anand NV (2009). Laparoscopic management of remnant cystic duct calculi: a retrospective study. Ann R Coll Surg Engl.

